# Longitudinal profiles of executive function in autistic and non-autistic children at high likelihood of autism

**DOI:** 10.1186/s11689-026-09682-4

**Published:** 2026-03-12

**Authors:** Tanya St. John, Neva M. Corrigan, Ariel Rokem, Annette M. Estes, Heather C. Hazlett, Juhi Pandey, Robert T. Schultz, Natasha Marrus, Lonnie Zwaigenbaum, Chimei M. Lee, Stephen R. Dager, Joseph Piven, IBIS network, J. Parish-Morris, B. Tunç, W. Guthrie, J. T. Elison, J. J. Wolff, M. D. Shen, J. B. Girault, R. Grzadzinski, A. M. Estes, D. Shaw, K. N. Botteron, R. C. McKinstry, J. N. Constantino, Alicia Rocca, J. C. Chappell, C. Burrows, M. R. Swanson, M. A. Styner, J. R. Pruett, A. C. Evans, L. C. MacIntyre, S. Torres-Gomez, S. Das, H. Volk, M. D. Fallin, G. Gerig

**Affiliations:** 1 Department of Speech and Hearing Science, University of Washington, Seattle, WA USA; 2 University of Washington Autism Center, University of Washington CHDD, Box 357920, Seattle, WA 98195 USA; 3 Institute On Human Development and Disability, University of Washington, Seattle, WA USA; 4 Institute for Learning and Brain Sciences, University of Washington, Seattle, WA USA; 5 Department of Psychology, University of Washington, Seattle, WA USA; 6 Carolina Institute for Developmental Disabilities, University of North Carolina at Chapel Hill, Chapel Hill, NC USA; 7 Department of Psychiatry, University of North Carolina at Chapel Hill School of Medicine, Chapel Hill, NC USA; 8 Center for Autism Research, Children’s Hospital of Philadelphia, Philadelphia, PA USA; 9 University of Pennsylvania Perelman School of Medicine, Philadelphia, PA USA; 10 Department of Psychiatry, Washington University School of Medicine in St. Louis, St. Louis, MO USA; 11 Department of Pediatrics, University of Alberta, Edmonton, AB Canada; 12 Department of Pediatrics, University of Minnesota, Minneapolis, MN USA; 13 Department of Radiology, University of Washington Medical Center, Seattle, WA USA

**Keywords:** Autism, Sibling, Executive function

## Abstract

**Background:**

Executive functioning (EF) difficulties are common in autistic children and may aggregate in families. Longitudinal studies examining EF development in autistic children and their siblings are limited. In the current study, we characterized EF development from later infancy through school-age in autistic and non-autistic high likelihood (HL; with an older autistic sibling) and low likelihood (LL; without older autistic sibling) children.

**Methods:**

Participants were 83 autistic and 235 non-autistic HL children, and 132 LL children from the Infant Brain Imaging Study. EF was assessed at ages 12 and 24 months using the A-not-B task and ages 5–14 years (school-age) using the Behavior Rating Inventory of Executive Function (BRIEF), Flanker Inhibitory Control and Attention Test (Flanker), and Dimensional Change Card Sort (DCCS). Longitudinal profiles of EF were examined using linear mixed-effects models with three separate models for each school-age EF outcome.

**Results:**

From 12–24 months, the LL group showed an increase in standardized EF scores (*p* < 0.05). The HL-ASD and HL-noASD groups showed no change (*ps* > .05). From 24 months to school-age, no change was observed for any group (*ps* > .05). From 12 months to school-age, standardized scores in the LL group increased (*p* = 0.002) and in the HL-ASD group (*p* < .001) decreased for the BRIEF GEC outcome. There were no group differences at 12 months in standardized EF scores (*ps* > .05) but at 24-months both HL groups had lower scores than the LL group (*p*s < .05). At school-age, the HL-ASD group had lower standardized EF scores than the LL and HL-noASD groups on all measures (*ps* < or = .050), and the HL-noASD group had lower standardized scores than the LL group on the BRIEF GEC (*p* = .014).

**Conclusions:**

Autistic and non-autistic HL children and LL children showed distinct EF profiles. On lab-based measures, autistic and non-autistic HL children showed relatively stable EF performance over time while LL children showed initial improvements. Parent report showed accumulating EF difficulties in autistic HL children. Executive function supports starting in toddlerhood may be useful for HL children showing emerging EF difficulties.

**Supplementary Information:**

The online version contains supplementary material available at 10.1186/s11689-026-09682-4.

Autism is a highly heritable neurodevelopmental condition characterized by difficulties with social communication and interaction and the presence of restricted and repetitive behaviors [1]. Though not a feature of autism according to current diagnostic criteria (DSM-5) [2], executive functioning (EF) difficulties are common and aggregate in families [3–6]. Younger siblings of autistic children (high likelihood; HL) are at increased likelihood of autism compared to younger siblings of non-autistic children (low likelihood; LL) and have a range of other developmental outcomes such as language delays, attention difficulties, and anxiety that may put them at risk for EF difficulties [7–11].

Executive functions, a diverse set of cognitive functions, support regulatory and goal directed behavior. Autistic children are nine times more likely to experience EF difficulties than non-autistic children, with observable difficulties, such as clinically elevated scores on standardized EF measures, by age 3 [12–15]. In autistic and non-autistic HL siblings—who are followed prospectively prior to the clinical presentation of autism—there is evidence of diverging EF by age 24 months [3]. However, longitudinal studies of EF in autism are limited. Additional longitudinal research is necessary to establish a comprehensive framework for EF development [16]. Such a framework could address current gaps in knowledge and inform the creation of more effective behavioral interventions and supports for autistic children.

EF develops rapidly in the first 3 years of life and continues to improve with age [17, 18]. But EF development may differ in autistic children. Studies show plateaued or worsening EF over time in autistic school-age children and adolescents [13, 16, 19–22], however, other studies showed improvements overtime in specific domains of EF [23–27]. Periods of increased growth may also be delayed. One study found rapid growth in EF (i.e., verbal working memory) during the last three years of elementary school, which was one year later than neurotypical children [28]. Although some studies report EF improvements in autistic children over time, lower scores on lab-based measures of EF or higher scores on informant rating scales of EF difficulties are still observed compared to neurotypical controls [23, 26, 27].

Existing research is constrained by reliance on either lab-based tests or parent-report rating scales of EF. While lab-based measures are regarded as offering a more objective assessment of executive abilities, they typically reflect optimal performance within a highly structured setting and may not correlate with real-world functioning [29]. Parent-report rating scales are considered more ecologically valid than lab-based measures because they provide an assessment of everyday use of executive functions and therefore may be more sensitive to detecting changes [24, 30]. However, parent-report measures may also be biased and thus, limited. Using both lab-based tests and rating scales of EF could provide an estimate of optimal performance as well as functioning in everyday life.

Current longitudinal studies of EF in autistic children cover limited developmental stages due to narrow age ranges and short intervals between timepoints, making it difficult to observe long term change or identify patterns across different developmental stages. For instance, many studies only tracked participants during the elementary school years and across intervals of 2–3 years. Only one study, to our knowledge, included autistic participants during later infancy through toddlerhood, finding that autistic and non-autistic HL children demonstrate slower growth in EF than LL children during this time [3]. Extending these findings beyond toddlerhood may provide further insights into the progression of EF across a broader range of developmental stages.

The predominance of clinically referred samples in longitudinal studies of EF in autistic children is another current limitation [16, 19, 20, 24, 27, 31]. Clinically referred samples may not reflect the larger population of autistic children, as they can be biased by those who seek clinical attention, which limits generalizability. Prospective research designs decrease this bias because participants are enrolled before clear developmental concerns or early signs of autism have emerged. Further, this approach allows for observation of executive functions in children prior to the onset of behavioral signs of autism or EF difficulties, as studies suggest that there are relevant EF predictors in infancy and toddlerhood that map on to later executive abilities [18]. This could allow for a better understanding of the mechanisms underlying the development of executive functions in autistic and HL children more broadly.

The main objective of the current study was to characterize EF development from later infancy through school-age in autistic and non-autistic high likelihood (HL; with an older autistic sibling) and low likelihood (LL; without older autistic sibling) children. We examined developmental profiles of EF using both lab-based measures and a parent rating scale. We also examined how sex, maternal education, and developmental functioning influenced these profiles [32–35].

## Methods

Participants were 318 HL and 132 LL children from the Infant Brain Imaging Study (IBIS), a prospective longitudinal study of brain and behavioral development in children at high and low likelihood of autism. IBIS methods have been detailed elsewhere, only study procedures relevant to the current project are described [36]. Study procedures were approved by each site’s Human Subjects Division, Institutional Review Board. Written informed consent was obtained from each participant’s parent. The LORIS data management platform was used for data collection, curation, preparation for analysis, and archiving [37].

Participants were recruited at four clinical sites: Children’s Hospital of Philadelphia, University of North Carolina, University of Washington, and Washington University in St. Louis. Older autistic siblings of HL participants were above the cut off for autism on the Social Communication Questionnaire (SCQ) and Autism Diagnostic Interview, Revised (ADI-R) [38, 39]. Older siblings of LL participants fell below the cut off score for autism on the SCQ and had no first-degree relative with intellectual disability or autism. Participants were screened at study entry for exclusionary criteria, which included: (1) birth weight < 2000 g and/or gestational age < 36 weeks or significant perinatal adversity and/or exposure in utero to neurotoxins, (2) medical/neurological conditions affecting growth, development, or cognition or significant sensory impairments, (3) known genetic conditions or syndromes, (4) adopted or half siblings, or twins, (5) first-degree relative with significant psychiatric conditions (e.g., schizophrenia), (6) contraindication for MRI and, (7) predominant home language other than English.

Participants were assessed at 12 and 24 months, and school age (5–14 years). At 12 and 24-months, assessments included developmental functioning, autism symptoms, and EF. School-age assessments included cognitive functioning, autism symptoms, and EF. School-age assessments were conducted over one or two days in a designated clinical or lab setting at each site. School-age EF measures were administered following cognitive and autism symptom assessment, either the same day or on a subsequent day, in a quiet environment with a qualified clinician. EF tests were delivered via iPads and in accordance with NIH Toolbox administration guidelines. Parents completed The Behavior Rating Inventory of Executive Function, Second Edition (BRIEF-2) online via LORIS as close to the in-person EF testing as possible [40]. A clinical best estimate diagnosis of autism spectrum disorder (ASD), available at 24 months, 36 months, or school age, was made by senior clinicians based on the Autism Diagnostic Observation Schedule, Generic and Second Edition (ADOS-G, ADOS-2) and ADI-R, and clinical judgement, using the Diagnostic and Statistical Manual of Mental Disorders, Fourth Edition Text Revision (DSM-IV-TR) criteria [39, 41–43]. Outcome groups were HL siblings who met ASD criteria (HL-ASD, *n* = 83), HL siblings who did not meet ASD criteria (HL-noASD, *n* = 235), and LL siblings who did not meet ASD criteria (LL, *n* = 132). Participant demographic and characteristic information is presented in Table 1.

**Table 1:** Participant demographics and characteristics

	**HL-ASD**	**HL-noASD**	**LL**
Total N	83	235	132
12-month visit			
Age (months)	12.7 (0.5)	12.8 (0.8)	12.8 (0.9)
Total N	27	80	44
24-month visit			
Age (months)	24.5 (0.6)	24.8 (0.9)	24.9 (1.1)
Total N	21	87	53
School-Age visit			
Age (months)	120.8 (19.1)	121.4 (21.5)	113.5 (21.0)
Total N	67	163	103
Sex (Male)	60 (72.3%)	132 (56.2%)	76 (57.6%)
Mother Education			
No college degree	31	67	21
College degree	52	167	111
Unknown/not reported	0	1	0
Race			
White	67	199	110
Asian	1	3	2
African-American	2	2	4
More than one race	12	30	14
Unknown/not reported	1	1	2
24-mo Mullen ELC	85.8 (17.6)	103.4 (16.1)	111.9 (15.5)
24-mo ADOS-2 Total CSS	5.2 (2.8)	1.4 (0.8)	1.4 (0.9)
School-Age DAS-II GCA	101.5 (21.7)	109.0 (14.2)	114.9 (13.4)
School-Age ADOS-2 Total CSS	5.6 (3.1)	2.0 (1.7)	1.5 (1.2)

ADOS-2 Total CSS, Autism Diagnostic Observation Schedule Total Calibrated Severity Score. Mullen ELC, Mullen Early Learning Composite. DAS-II GCA, Differential Ability Scale General Conceptual Ability. Flanker, Flanker Inhibitory Control and Attention Test. DCCS, Dimensional Change Card Sort. GEC, Global Executive Composite

*HL-ASD* High likelihood diagnosed with autism spectrum disorder, *HL-noASD* High-likelihood not diagnosed with autism, *LL* Low likelihood

## Measures

### Executive function

The A-not-B task was administered at 12 and 24-months. The A-not-B assesses EF (i.e., working memory, response inhibition, and cognitive flexibility) in infancy through preschool [44–46]. In this task, children are required to maintain a mental representation of an object’s location while it is hidden, inhibit a previously learned response when the object is moved to a new location, and adapt to the switch in hiding location. Children watched as a toy was hidden to the left or right of midline in a well and were encouraged to find the toy after a 5 s delay. Once the hidden toy was found on two consecutive trials, the side of hiding was reversed and continued in this pattern until three sets of correct responses at 5 s delay were successfully completed. The delay was then increased to 12 s. A maximum of 24 trials and 4 reversal trials were administered. Performance was measured by the proportion of total correct reaches by total trials.

The Behavior Rating Inventory of Executive Function, Second Edition (BRIEF-2), administered at school-age, is a 63-item parent report measure that assesses EF difficulties in children ages 5–18 years [40]. The BRIEF-2, rated on a 3-point Likert scale, is comprised of 9 clinical scales (Inhibit, Self-Monitor, Shift, Emotional Control, Initiate, Working Memory, Plan/Organize, Task Monitor, and Organization of Materials) and yields three domain indices (Behavior Regulation Index, Emotion Regulation Index, and Cognitive Regulation Index) and an overall Global Executive Composite (GEC) standard score. Twenty-one participants received the original version of the BRIEF (HL-ASD = 7, HL-noASD = 6, LL = 8), which is highly correlated (*r* ≥ 0.80) with the BRIEF-2 [40, 47]. Scores are reported as *T* scores, which have a mean of 50 and have a standard deviation of 10. Higher scores represent increased EF difficulties. The Global Executive Composite and Working Memory, Inhibit, and Shift subscales were used in this study. We chose these specific subscales because they align with the basic executive functions assessed by the A-not-B.

The Flanker Inhibitory Control and Attention Test (Flanker) and Dimensional Change Card Sort (DCCS) are iPad tasks for children ages 4 + years and were administered at school-age [48]. The Flanker is an assessment of inhibitory control and attention. The child is asked to focus on a central stimulus (arrow or fish) while inhibiting attention to the stimuli surrounding it (arrows or fish pointing in the same or different direction as the central stimulus). The DCCS is an assessment of cognitive flexibility and attention where the child is asked to sort a series of target pictures first according to one dimension then another. The child is presented with a pair of response choices that matches one of these dimensions, however, the dimension by which the child matches the target to the picture pairs changes intermittently, requiring the child to shift their responses to get the correct answer. The Flanker and DCCS yield a Ratio of Correct responses to Speed (RCS) score which are converted to an age-normed, standard score; higher scores represent better performance.

### Developmental level

The Mullen Scales of Early Learning (MSEL) is a standardized, normed, developmental assessment for children from birth through 68 months of age. The MSEL consists of five scales that assess early development (Visual Reception, Expressive and Receptive Language, Gross and Fine Motor) with a composite standard score, the Early Learning Composite Standard Score (ELC), used as a measure of overall developmental level.

#### Quality control

Executive functioning data were visually inspected for normality and outliers using histograms and residual plots. No multivariate outliers were identified.

#### Statistical analysis

Longitudinal changes in EF were examined using linear mixed-effects (LME) models implemented in R (v. 4.4.2). The three timepoints (12 months, 24 months and school-age) were modeled as categorical factors. Fixed effects were sex, diagnostic group, timepoint and group x time interaction. Participant identifiers were included as random intercepts to account for repeated measures within subjects. The LL group and the 12-month timepoint were used as reference categories. Model estimates were obtained using restricted maximum likelihood. Planned pairwise contrasts for the group x time interaction were conducted using estimated marginal means. These planned comparison results were not corrected for multiple comparisons.

Three separate models were fit due to the availability of three different school-age EF measures (Flanker, DCCS, and BRIEF GEC). Each model incorporated the same 12- and 24-month EF data, along with one school-age EF outcome measure. All EF scores were converted to Z-scores prior to inclusion in the models, using the mean and standard deviation of the full sample at each age point. BRIEF Z-scores were sign-reversed so that higher scores indicate better EF, consistent with scores for the other EF measures.

This modeling was repeated with maternal education and developmental level added as covariates. Developmental level was represented as a Z-score derived from the Mullen Scale of Early Learning composite score at 24 months. This timepoint for developmental level was selected because it contained the least amount of missing data—as compared to the Mullen scores at 12 months and Differential Ability Scale, Second Edition (DAS-II) scores at school age—allowing for data maximization. Participants with missing maternal education or developmental data were excluded from these covariate-adjusted analyses.

## Results

### EF Profiles

The results from the LME models are displayed in Tables 2, 3, 4 and 5. Profiles of EF across time are displayed in Figs. 1, 2, and 3. Models with covariates and profiles for secondary analyses are provided in supplemental materials (Table S1 and S2, Figures S1-6).

**Table 2:** Longitudinal model of executive functioning by school-age outcome

	**Flanker**			**DCCS**			**BRIEF**		
**Term**	***β***	**SE**	***p***	***β***	**SE**	***p***	***β***	**SE**	***P***
(Intercept)	−0.12	0.16	0.435	−0.06	0.16	0.720	−0.05	0.15	0.731
Sex: Male	0.01	0.08	0.951	−0.12	0.08	0.154	−0.12	0.08	0.122
Group: HL-noASD^a^	0.22	0.18	0.241	0.21	0.18	0.266	0.20	0.18	0.243
Group: HL-ASD^a^	0.04	0.24	0.856	0.07	0.24	0.766	0.06	0.23	0.785
Time: 24 months^b^	0.48	0.20	0.014	0.47	0.20	0.019	0.47	0.19	0.019
Time: school-age^b^	0.30	0.18	0.090	0.26	0.18	0.145	0.53	0.17	0.007
Group: HL-noASD*Time: 24 months^ab^	−0.66	0.25	0.008	−0.64	0.25	0.010	−0.64	0.24	0.005
Group: HL-ASD*Time: 24 months^ab^	−0.95	0.34	0.006	−0.94	0.35	0.007	−0.93	0.33	0.005
Group: HL-noASD*Time: school-age^ab^	−0.38	0.22	0.089	−0.32	0.23	0.162	−0.52	0.21	0.016
Group: HL-ASD*Time: school-age^ab^	−0.56	0.29	0.053	−0.49	0.29	0.095	−1.38	0.28	< .001

*HL-ASD* High likelihood diagnosed with autism, *HL-noASD* High-likelihood not diagnosed with autism, *LL* Low likelihood, *BRIEF GEC* Behavior Rating Inventory of Executive Function Global Executive Composite, *Flanker* Flanker Inhibitory Control and Attention Test, *DCCS* Dimensional Change Card Sort

^a^Reference category is LL

^b^Reference category is 12-month Time point

**Fig. 1:**
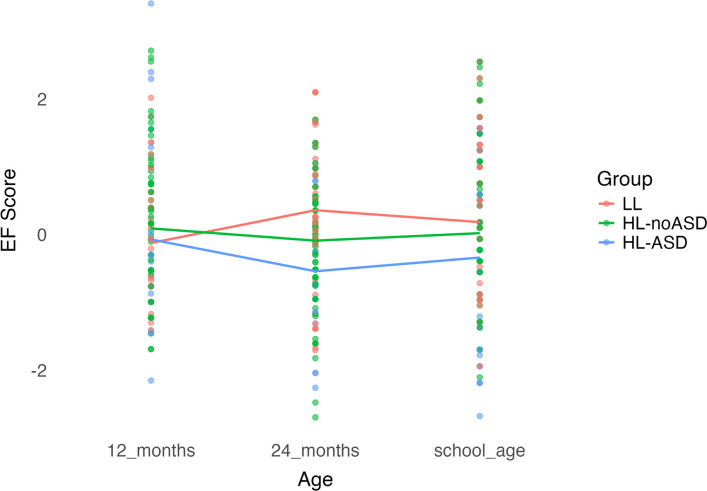
Longitudinal profiles of executive functioning with Flanker as outcome

**Fig. 2:**
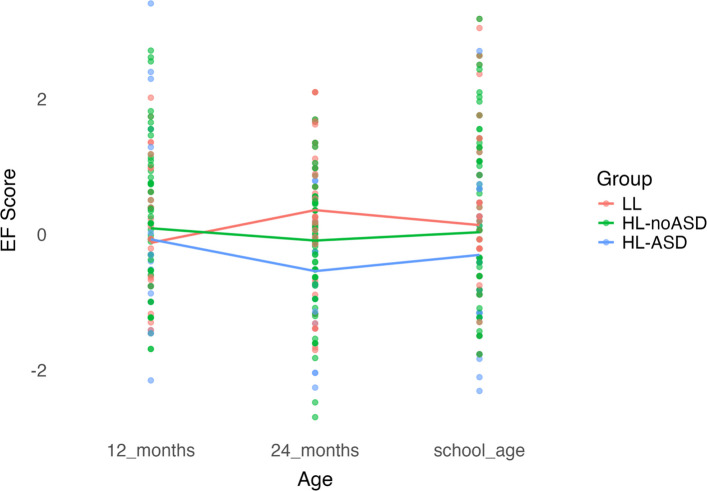
Longitudinal profiles of executive functioning with DCCS as outcome

**Fig. 3:**
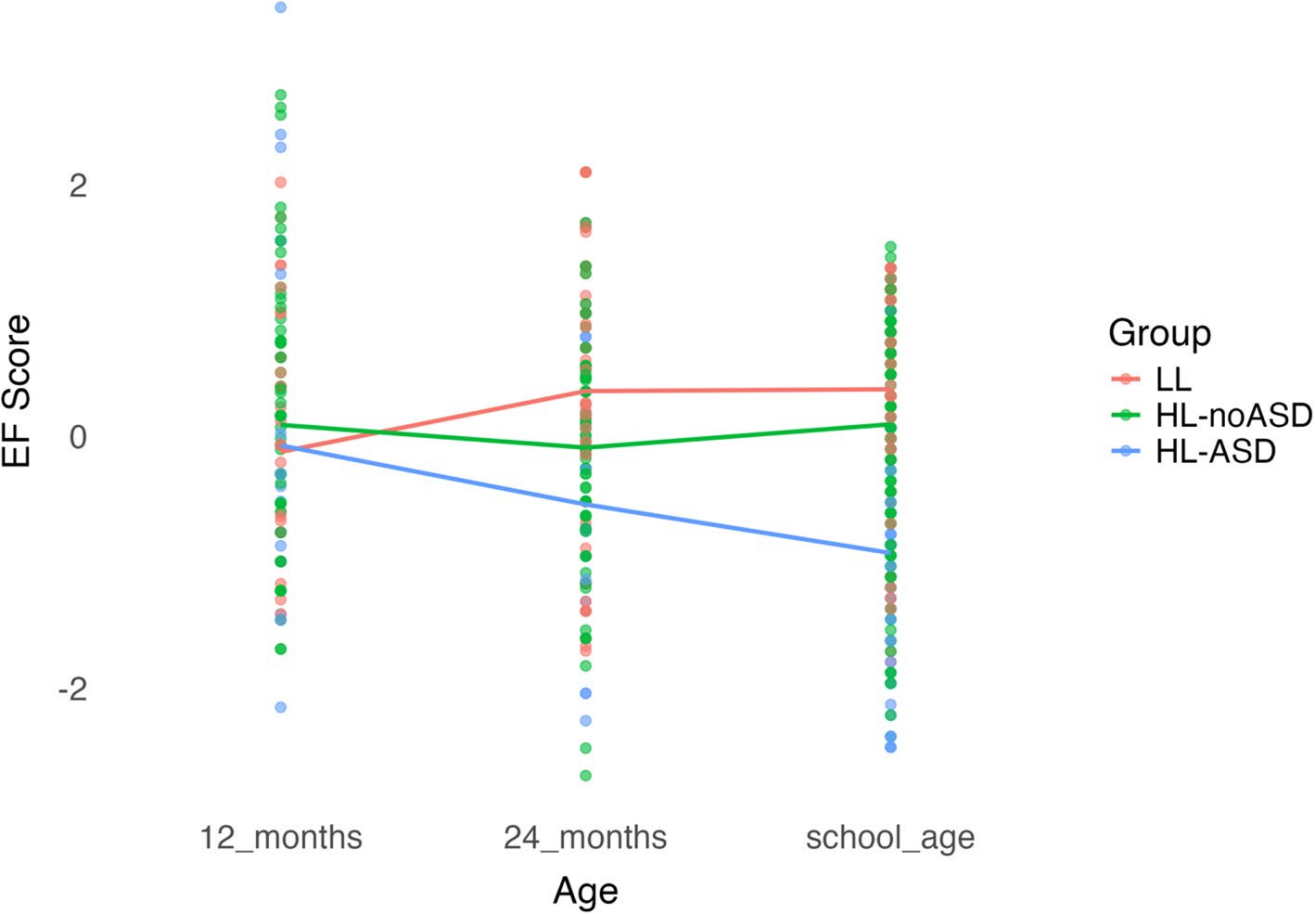
Longitudinal profiles of executive functioning with BRIEF as outcome

Group x time interactions were significant at 24 months in all models, regardless of school-age measure, and at school-age when the BRIEF GEC was used as the school-age measure (Table 2). From 12–24 months, the HL-ASD and HL-noASD groups showed a decrease in standardized EF scores relative to the LL group, as indicated by significant negative group x time interactions ( *p* ≤ 0.010, *p* ≤ 0.007, respectively) whereas the LL group showed a significant increase in standardized EF scores over the same period (*p* ≤ 0.019). From 12 months to school-age, the HL-ASD group showed a decrease in standardized EF scores relative to the LL group that was significant for the BRIEF GEC (*p* < 0.001) and non-significant on the Flanker (*p* = 0.053) and DCCS (*p* = 0.095). The HL-noASD group also showed a decrease in standardized EF scores from 12 months to school-age relative to the LL group that were significant for the BRIEF GEC (*p* = 0.016) and non-significant for the Flanker (*p* = 0.089) and DCCS (*p* = 0.162). The LL group showed an increase in standardized EF scores from 12 months to school-age that were significant for the BRIEF GEC (*p* = 0.007) and non-significant for the Flanker (*p* = 0.090) and DCCS (*p* = 0.145). When maternal education and 24-month developmental level were added as covariates in the models, the HL-ASD group showed a significant decrease in standardized EF scores from 12 months to school-age relative to the LL group for the Flanker (*p* = 0.044; Table S1). There were no other changes to statistical significance or directionality of slopes.

Planned comparisons using estimated marginal means for group at each age point (Table 3) showed no significant group differences in EF at 12 months (*ps* > 0.05). At 24-months, the HL-ASD and HL-noASD groups had significantly lower standardized EF scores than the LL group (*p*s ≤ 0.011) but standardized scores of the two groups did not differ significantly from each other. At school-age, the HL-ASD group had significantly lower standardized EF scores than the LL group on the BRIEF GEC (*p* < 0.001), Flanker (*p* = 0.002), and DCCS (*p* = 0.013). The HL-ASD group also had significantly lower standardized scores on the BRIEF GEC, Flanker, and DCCS than the HL-noASD group (*p* < 0.001, *p* = 0.022, *p* = 0.050, respectively). The HL-noASD group had significantly lower standardized scores than the LL group on the BRIEF GEC (*p* = 0.014) but did not differ from the LL group on the Flanker and DCCS (*p* = 0.210, *p* = 0.404, respectively). When controlling for maternal education and 24-month developmental level, the difference in standardized scores between the HL-noASD and HL-ASD group on the Flanker, all group differences on the DCCS, and the difference between LL and HL-noASD on the BRIEF GEC became non-significant (Table S2).

**Table 3:** Estimated marginal means time and group

	**Flanker**			**DCCS**			**BRIEF**		
**Contrast**	**Estimate**	**SE**	***p***	**Estimate**	**SE**	***p***	**Estimate**	**SE**	***P***
12 months									
LL—HL-noASD	−0.22	0.19	0.242	−0.21	0.19	0.267	−0.20	0.18	0.244
LL—HL-ASD	−0.04	0.24	0.857	−0.07	0.24	0.766	−0.06	0.23	0.785
HL-noASD—HL-ASD	0.17	0.22	0.432	0.13	0.22	0.544	0.14	0.21	0.496
24 months									
LL—HL-noASD	0.45	0.17	0.010	0.44	0.17	0.011	0.44	0.16	0.008
LL—HL-ASD	0.91	0.26	< .001	0.87	0.26	0.001	0.87	0.24	< .001
HL-noASD—HL-ASD	0.46	0.24	0.054	0.43	0.24	0.072	0.43	0.23	0.059
School age									
LL—HL-noASD	0.16	0.13	0.210	0.11	0.13	0.404	0.31	0.13	0.014
LL—HL-ASD	0.51	0.16	0.002	0.41	0.17	0.013	1.32	0.16	< .001
HL-noASD—HL-ASD	0.35	0.15	0.022	0.30	0.15	0.050	1.00	0.15	< .001
LL									
12 months—24 months	−0.48	0.20	0.014	−0.47	0.20	0.019	−0.47	0.19	0.014
12 months—school age	−0.30	0.18	0.091	−0.26	0.18	0.146	−0.53	0.17	0.002
24 months—school age	0.18	0.17	0.276	0.21	0.17	0.224	−0.06	0.16	0.691
HL-noASD									
12 months—24 months	0.18	0.15	0.232	0.18	0.15	0.248	0.17	0.14	0.227
12 months—school age	0.08	0.14	0.560	0.05	0.14	0.692	−0.01	0.13	0.924
24 months—school age	−0.10	0.13	0.447	−0.12	0.13	0.365	−0.19	0.13	0.143
HL-ASD									
12 months—24 months	0.47	0.28	0.096	0.48	0.28	0.096	0.46	0.27	0.086
12 months—school age	0.26	0.23	0.259	0.22	0.23	0.328	0.85	0.22	< .001
24 months—school age	−0.21	0.25	0.386	−0.25	0.25	0.316	0.39	0.24	0.108

*HL-ASD* High likelihood diagnosed with autism, *HL-noASD* High-likelihood not diagnosed with autism, *LL* Low likelihood, *BRIEF GEC* Behavior Rating Inventory of Executive Function Global Executive Composite, *Flanker* Flanker Inhibitory Control and Attention Test, *DCCS* Dimensional Change Card Sort

Planned comparisons using estimated marginal means for time (Table 3) showed no difference in standardized EF scores from 12–24 months for both the HL-ASD and HL-noASD groups (*ps* > 0.05), and a significant increase in standardized EF scores for the LL group (*p* ≤ 0.019). From 24 months to school-age, changes in standardized EF scores were non-significant across all groups for the Flanker, DCCS, and BRIEF GEC (*ps* > 0.05). From 12 months to school-age, standardized EF scores for the Flanker and DCCS did not change significantly for any group (*p*s > 0.05). However, there was a significant increase in standardized EF scores from 12 months to school age in the LL group (*p* = 0.002) and significant decrease in standardized EF scores in the HL-ASD group (*p* < 0.001) for the BRIEF GEC. The findings did not change when controlling for maternal education and 24-month developmental level (Table S2).

A secondary analysis examined the three BRIEF subscales—Working Memory, Inhibit, and Shift—that align with executive functions assessed by the A-not-B. Group x time interactions were significant for all three BRIEF subscales at 24 months and at school-age (Table 4.). Results for 12–24 months across all BRIEF subscales were similar to that of the BRIEF GEC. From 12 months to school-age, the HL-ASD group showed a decrease in standardized EF scores relative to the LL group for all BRIEF subscales (*ps* < 0.001). The HL-noASD group also showed a decrease in standardized EF scores from 12 months to school-age relative to the LL group that were significant for Working Memory (*p* = 0.009) and Inhibit (*p* = 0.048) subscales and non-significant for the Shift subscale (*p* = 0.104). The LL group showed an increase in standardized EF scores from 12 months to school-age that were significant for all BRIEF subscales (*ps* < 0.010). Planned comparisons using estimated marginal means for group at each age point (Table 5) showed that at school-age, the HL-ASD had significantly lower standardized EF scores than the LL and HL-noASD groups on all BRIEF subscales (*ps* < 0.001). The HL-noASD group had significantly lower standardized scores on the Working Memory subscale (*p* = 0.005) than the LL group. Planned comparisons using estimated marginal means for time (Table 5) showed a significant increase for the HL-noASD (*p* = 0.025) and significant decrease for the HL-ASD group (*p* = 0.043) in standardized EF scores for the Shift subscale from 24 months to school-age. From 12 months to school-age, there was a significant increase in standardized EF scores in the LL group (*ps* ≤ 0.008) and significant decrease in the HL-ASD group (*p* ≤ 0.002) for all three BRIEF subscales.

**Table 4:** Longitudinal model of executive functioning by BRIEF subscale

**Working Memory**	**Working Memory**			**IOnhibit**			**Shift**		
**Term**	***b***	**SE**	***p***	***b***	**SE**	***p***	***b***	**SE**	***p***
(Intercept)	-0.08	0.15	0.581	-0.06	0.15	0.705	-0.04	0.15	0.778
Sex: Male	-0.07	0.08	0.394	-0.12	0.08	0.136	-0.13	0.08	0.101
Group: HL-noASD	0.21	0.18	0.242	0.21	0.18	0.240	0.20	0.17	0.251
Group: HL-ASD	0.06	0.23	0.809	0.06	0.23	0.780	0.05	0.23	0.827
Time: 24 months	0.47	0.19	0.014	0.47	0.19	0.013	0.46	0.18	0.012
Time: school-age	0.52	0.17	0.003	0.46	0.17	0.007	0.45	0.17	0.008
Group: HL-noASD*Time: 24 months	-0.65	0.24	0.008	-0.65	0.24	0.007	-0.64	0.23	0.006
Group: HL-ASD*Time: 24 months	-0.94	0.34	0.005	-0.94	0.33	0.005	-0.92	0.32	0.004
Group: HL-noASD*Time: school-age	-0.58	0.22	0.009	-0.43	0.22	0.048	-0.34	0.21	0.104
Group: HL-ASD*Time: school-age	-1.21	0.28	< .001	-1.25	0.28	< .001	-1.38	0.27	< .001

*HL-ASD* High likelihood diagnosed with autism, *HL-noASD* High-likelihood not diagnosed with autism, *LL* Low likelihood

^a^Reference category is LL

^b^Reference category is 12-month Time point

**Table 5:** Estimated marginal means time and group for BRIEF subscales

	**Working Memory**			**Inhibit**			**Shift**		
**Contrast**	**Estimate**	**SE**	***p***	**Estimate**	**SE**	***p***	**Estimate**	**SE**	***P***
12 months									
LL—HL-noASD	−0.21	0.18	0.243	−0.21	0.18	0.242	−0.20	0.17	0.252
LL—HL-ASD	−0.06	0.23	0.809	−0.06	0.23	0.780	−0.05	0.23	0.828
HL-noASD—HL-ASD	0.15	0.21	0.473	0.14	0.21	0.497	0.15	0.21	0.469
24 months									
LL—HL-noASD	0.44	0.17	0.008	0.44	0.17	0.008	0.44	0.16	0.006
LL—HL-ASD	0.88	0.25	< .001	0.87	0.24	< .001	0.87	0.24	< .001
HL-noASD—HL-ASD	0.44	0.23	0.058	0.43	0.23	0.060	0.43	0.23	0.057
School age									
LL—HL-noASD	0.37	0.13	0.005	0.22	0.13	0.086	0.14	0.13	0.250
LL—HL-ASD	1.15	0.16	< .001	1.18	0.16	< .001	1.33	0.16	< .001
HL-noASD—HL-ASD	0.78	0.15	< .001	0.96	0.15	< .001	1.19	0.15	< .001
LL									
12 months—24 months	−0.47	0.19	0.014	−0.47	0.19	0.013	−0.46	0.18	0.012
12 months—school age	−0.52	0.17	0.003	−0.46	0.17	0.007	−0.45	0.17	0.008
24 months—school age	−0.05	0.16	0.756	0.01	0.16	0.970	0.02	0.16	0.908
HL-noASD									
12 months—24 months	0.18	0.15	0.231	0.17	0.14	0.228	0.18	0.14	0.209
12 months—school age	0.05	0.13	0.698	−0.04	0.13	0.781	−0.10	0.13	0.424
24 months—school age	−0.12	0.13	0.339	−0.21	0.13	0.100	−0.28	0.13	0.025
HL-ASD									
12 months—24 months	0.46	0.28	0.092	0.47	0.27	0.086	0.46	0.26	0.084
12 months—school age	0.68	0.22	0.002	0.78	0.22	< .001	0.94	0.22	< .001
24 months—school age	0.22	0.24	0.374	0.32	0.24	0.189	0.48	0.24	0.043

*HL-ASD* High likelihood diagnosed with autism, *HL-noASD* High-likelihood not diagnosed with autism, *LL* Low likelihood

^a^Reference category is LL

^b^Reference category is 12-month Time point

## Discussion

We found distinct EF profiles for autistic and non-autistic HL and LL children. We examined standardized EF scores at 12 and 24 months of age and school-age. At school-age, three distinct measures captured different aspects of EF, revealing unique patterns across the three groups. When measures at all timepoints were lab-based (i.e. A-not-B, Flanker, DCCS), autistic and non-autistic HL children showed relatively stable EF performance over time, while LL children showed improving then stable performance. In the mixed-measure analysis, where lab-based measures were used at 12 and 24 months and parent-report at school-age, a different pattern emerged. Autistic HL children showed decreasing standardized EF scores across time, while non-autistic HL children remained stable and LL children improved.

In alignment with previous studies, we found that autistic HL children had reduced EF performance on lab-based measures and higher parent-reported EF difficulties than LL children [23, 26, 27, 49]. Non-autistic HL children also had reduced performance at 24 months but similar performance on lab-based measures and higher parent-reported EF difficulties at school-age compared to LL children. EF differences between autistic HL children and LL children were greatest at school-age, while for non-autistic HL children the largest gap appeared at 24 months. Further, standardized EF scores remained relatively consistent across all age intervals (12–24 months, 24 months to school-age, 12 months to school-age) for both autistic and non-autistic HL children, suggesting stable performance over time. In contrast, LL children’s performance improved from 12 to 24 months and remained stable from 24 months to school-age. In the mixed-measure analysis, we found the biggest changes in standardized scores from 12 months to school-age in autistic HL children, perhaps representing an accumulation of EF difficulties in this group.

When we performed secondary analyses of BRIEF subscales corresponding to executive functions assessed by the A-not-B task, we found that autistic HL children had greater parent-reported working memory, inhibition, and cognitive flexibility difficulties at school-age than both non-autistic HL and LL children. Further, non-autistic HL children had greater parent-reported working memory difficulties than LL children but not greater inhibition or cognitive flexibility difficulties. Unlike the composite BRIEF score, standardized EF scores for the cognitive flexibility outcome showed marked changes between 24-months and school-age in both autistic and non-autistic HL children, but in opposite directions: autistic HL children exhibited greater difficulties in EF while non-autistic HL children showed improvement. This pattern may be directly associated with the clinical features of autism, which often involve cognitive flexibility challenges.

EF profiles varied by school-age outcome measure type in our study. When using models that included lab-based measures (Flanker and DCCS) at the school-age timepoint, a pattern of relatively stable EF performance over time was observed. The mixed-measure approach, where the school-age measure was a rating scale of parent-reported EF difficulties (BRIEF) covering a broad range of executive functions, significant change over time was observed. Prior studies using rating scales of EF difficulties show a similar pattern of increasing difficulties in autistic children over time, while studies using lab-based measures tend to find flat trajectories [13, 16, 19–22]. Differences in findings across measure types may reflect differences between EF capacity assessed with lab-based measures in a specific context versus day-to-day implementation of EF skills, which is captured by parent-report. If true, this could suggest that despite capacity, autistic children experience challenges in applying their EF abilities to everyday situations. These differences may also be a result of lab-based measures capturing behavior at a single timepoint, while parent report reflect EF skills in aggregate over longer periods.

This study is the first, to our knowledge, to examine developmental profiles of EF in autistic and non-autistic HL children from infancy to school-age. We found qualitatively different patterns of EF across time that differed by group. Diverging EF profiles among HL children could reflect variance in clinical outcomes above and beyond autism. For example, a recent study of toddlers at HL of autism and attention deficit hyperactivity disorder (ADHD) found that different EF patterns were associated with autism versus ADHD characteristics [50]. While not addressed in the current study, future research could examine the interaction between long-term developmental patterns of EF, autism, and other clinical outcomes such as ADHD and anxiety, given that these co-occurring conditions are observed at higher rates in HL children [11, 51–53]. Further investigation into patterns of cognitive inflexibility in autistic and non-autistic HL children is also warranted to understand their contribution to co-occurring conditions. These patterns could play a role in the development or exacerbation of emotional difficulties [54].

It is important to acknowledge certain limitations of this study. Due to the extensive age range of our sample (12 months to school-age), it was necessary to use developmentally sensitive measures of EF, which varied over time. As a result, our measure of executive function at 12 and 24 months of age differed from our measures at school-age. Measurement change is a significant hurdle in longitudinal research, particularly in studies examining EF over a wide range of developmental stages. We addressed this challenge by standardizing scores across all EF measures though this approach is not universal and may not represent true growth but rather relative performance [25]. Because the school-age group included children from 5 to 14 years old, there may be considerable developmental heterogeneity at this timepoint. This could result in measurement of qualitatively different abilities across different ages despite using the same measure for all participants at this timepoint, potentially obscuring age-specific developmental patterns and raising some uncertainty as to whether scores reflect the same executive processes across participants. Additionally, we examined EF profiles using group means, though individual trajectories could provide higher-level information for more targeted, individualized intervention approaches and should be considered with larger samples. Our findings suggest that executive function supports may be useful starting as early as 24 months of age for HL children showing emerging difficulties in EF. Further, preschool is generally considered a period of significant EF growth; however, this developmental stage was not specifically included in our study but should be considered in future research given its importance to EF development [55]. Comparing developmental profiles of our sample to children with non-autism neurodevelopmental conditions and EF difficulties could also provide greater specificity to our findings. Finally, given the novelty of the data, there was no correction for multiple comparisons in our planned comparison analyses, which may have inflated type I error; therefore, replication of these findings with a larger HL sample would be an important next step. Replication in a sample of non-English speakers would also enhance the generalizability of the findings.

This study has several strengths. For the first time, to our knowledge, we demonstrate in a longitudinal sample that EF difficulties in autistic children span from later infancy to school-age. We were able to observe long term changes in EF that spanned several developmental stages due to our broad age range (12 months to school-age) and longer interval across timepoints (span of over 10 years). The use of a prospective design with an unbiased, well-characterized sample of autistic children was another strength, that collectively with studies using clinically referred samples, increases generalizability to the broader autistic population. Lastly, we used both lab-based measures and a parent-reported rating scale of EF, which balanced potential tendencies of lab-based measures to demonstrate optimal performance with potential biases of parent-report measures.

We found that autistic and non-autistic HL children and LL children showed distinct EF profiles that began in later infancy and persisted through school-age. This study underscores the value of integrating multiple sources of information (e.g., measures of direct performance and parent report) to enhance our understanding of EF patterns over time, helping build a clearer framework for EF development in autistic children and those at increased likelihood of neurodevelopmental conditions. Ultimately, these findings may support the design of more effective behavioral interventions and supports for autistic children, for example, by underscoring the importance of targeted support for EF development in autistic children particularly during the period between toddlerhood and school-age.

## Electronic Supplementary Material

Below is the link to the electronic supplementary material.

Supplementary Material 1

## Data Availability

The datasets used and analyzed in the current study can be made available by the corresponding author upon reasonable request, and subject to IRB approval. Data for this study is included in the National Institute of Mental Health Data Archive (NDA) https://nda.nih.gov/.

## Abbreviations

BRIEF: Behavior Rating Inventory of Executive Function
DSM-IV: Diagnostic and Statistical Manual of Mental Disorders, Fourth Edition
DCCS: Dimensional Change Card Sort
Flanker: Flanker Inhibitory Control and Attention Task
GEC: Global Executive Composite
HL: High Familial Likelihood for Autism
HL-noASD: High Familial Likelihood for Autism and No Autism Diagnosis
HL-ASD: High Familial Likelihood for Autism and Autism Diagnosis
IBIS: Infant Brain Imaging Study
LL: Low Familial Likelihood, No Autism Diagnosis
