# Supplementary material for: Longitudinal profiles of executive function in autistic and non-autistic children at high likelihood of autism
Source: J Neurodev Disord. 2026 Mar 12;18:21. doi: 10.1186/s11689-026-09682-4 (PMC13093929; doi:10.1186/s11689-026-09682-4)

Table S1. Longitudinal Model of Executive Functioning by School-age Outcome with Covariates

|  | **Flanker** | | | |  | | **DCCS** | | | |  | | **BRIEF** | | | |
| --- | --- | --- | --- | --- | --- | --- | --- | --- | --- | --- | --- | --- | --- | --- | --- | --- |
| **Term** | ***β*** | **SE** | ***p*** |  | | ***β*** | | **SE** | ***p*** |  | | ***β*** | | **SE** | ***p*** |  |
| (Intercept) | -0.18 | 0.18 | 0.306 |  | | -0.07 | | 0.18 | 0.674 |  | | -0.19 | | 0.17 | 0.265 |  |
| Sex: Male | 0.01 | 0.09 | 0.868 |  | | -0.11 | | 0.09 | 0.206 |  | | -0.08 | | 0.08 | 0.312 |  |
| 24 mo Mullen ELC | 0.09 | 0.05 | 0.065 |  | | 0.11 | | 0.05 | **0.035** |  | | 0.13 | | 0.05 | **0.007** |  |
| Maternal Education | -0.01 | 0.10 | 0.920 |  | | -0.07 | | 0.10 | 0.498 |  | | 0.04 | | 0.09 | 0.672 |  |
| Group: HL-noASD | 0.28 | 0.19 | 0.139 |  | | 0.26 | | 0.19 | 0.167 |  | | 0.29 | | 0.18 | 0.107 |  |
| Group: HL-ASD | 0.18 | 0.25 | 0.472 |  | | 0.21 | | 0.25 | 0.404 |  | | 0.25 | | 0.24 | 0.289 |  |
| Time: 24 months | 0.50 | 0.20 | **0.012** |  | | 0.48 | | 0.20 | **0.017** |  | | 0.48 | | 0.19 | **0.012** |  |
| Time: school-age | 0.33 | 0.18 | 0.065 |  | | 0.27 | | 0.18 | 0.132 |  | | 0.51 | | 0.17 | **0.003** |  |
| Group: HL-noASD*Time: 24 months | -0.69 | 0.25 | **0.006** |  | | -0.66 | | 0.25 | **0.009** |  | | -0.66 | | 0.24 | **0.006** |  |
| Group: HL-ASD*Time: 24 months | -0.98 | 0.34 | **0.005** |  | | -0.97 | | 0.35 | **0.006** |  | | -0.96 | | 0.33 | **0.004** |  |
| Group: HL-noASD*Time: school-age | -0.44 | 0.23 | 0.055 |  | | -0.33 | | 0.23 | 0.145 |  | | -0.49 | | 0.22 | **0.025** |  |
| Group: HL-ASD*Time: school-age | -0.59 | 0.29 | **0.044** |  | | -0.50 | | 0.29 | 0.087 |  | | -1.36 | | 0.28 | **0.000** |  |

*Note*. HL-ASD, high likelihood diagnosed with autism; HL-noASD, high-likelihood not diagnosed with autism; LL, low likelihood. ^a^ Reference category is LL. ^b^ Reference category is 12-month Time point. Mullen ELC, Mullen Early Learning Composite. BRIEF GEC, Behavior Rating Inventory of Executive Function Global Executive Composite. Flanker, Flanker Inhibitory Control and Attention Test. DCCS, Dimensional Change Card Sort.

Table S2. Estimated Marginal Means Time and Group with Covariates

|  |  | **Flanker** |  |  |  | **DCCS** |  |  |  | **BRIEF** |  |  |
| --- | --- | --- | --- | --- | --- | --- | --- | --- | --- | --- | --- | --- |
| **Contrast** |  | **Estimate** | **SE** | ***p*** |  | **Estimate** | **SE** | ***p*** |  | **Estimate** | **SE** | ***p*** |
| **12 months** |  |  |  |  |  |  |  |  |  |  |  |  |
| LL - HL-noASD | | -0.28 | 0.19 | 0.140 |  | -0.26 | 0.19 | 0.168 |  | -0.29 | 0.18 | 0.108 |
| LL - HL-ASD | | -0.18 | 0.25 | 0.473 |  | -0.21 | 0.25 | 0.405 |  | -0.25 | 0.24 | 0.290 |
| HL-noASD - HL-ASD | | 0.10 | 0.23 | 0.668 |  | 0.05 | 0.23 | 0.823 |  | 0.03 | 0.21 | 0.871 |
| **24 months** |  |  |  |  |  |  |  |  |  |  |  |  |
| LL - HL-noASD | | 0.41 | 0.17 | **0.019** |  | 0.40 | 0.17 | **0.021** |  | 0.38 | 0.16 | **0.022** |
| LL - HL-ASD | | 0.80 | 0.26 | **0.002** |  | 0.76 | 0.26 | **0.004** |  | 0.71 | 0.25 | **0.004** |
| HL-noASD - HL-ASD | | 0.39 | 0.24 | 0.106 |  | 0.36 | 0.24 | 0.143 |  | 0.33 | 0.23 | 0.149 |
| **School age** |  |  |  |  |  |  |  |  |  |  |  |  |
| LL - HL-noASD | | 0.16 | 0.14 | 0.248 |  | 0.07 | 0.14 | 0.590 |  | 0.20 | 0.13 | 0.124 |
| LL - HL-ASD | | 0.40 | 0.18 | **0.023** |  | 0.29 | 0.18 | 0.100 |  | 1.11 | 0.18 | **< .001** |
| HL-noASD - HL-ASD | | 0.25 | 0.16 | 0.119 |  | 0.22 | 0.16 | 0.169 |  | 0.91 | 0.16 | **< .001** |
| **LL** |  |  |  |  |  |  |  |  |  |  |  |  |
| 12 months - 24 months | | -0.50 | 0.20 | **0.012** |  | -0.48 | 0.20 | **0.017** |  | -0.48 | 0.19 | **0.012** |
| 12 months - school age | | -0.33 | 0.18 | 0.066 |  | -0.27 | 0.18 | 0.133 |  | -0.51 | 0.17 | **0.003** |
| 24 months - school age | | 0.17 | 0.17 | 0.323 |  | 0.20 | 0.17 | 0.231 |  | -0.03 | 0.16 | 0.838 |
| **HL-noASD** |  |  |  |  |  |  |  |  |  |  |  |  |
| 12 months - 24 months | | 0.19 | 0.15 | 0.216 |  | 0.19 | 0.15 | 0.227 |  | 0.18 | 0.15 | 0.207 |
| 12 months - school age | | 0.10 | 0.14 | 0.452 |  | 0.06 | 0.14 | 0.664 |  | -0.02 | 0.13 | 0.862 |
| 24 months - school age | | -0.08 | 0.13 | 0.529 |  | -0.12 | 0.13 | 0.355 |  | -0.21 | 0.13 | 0.109 |
| **HL-ASD** |  |  |  |  |  |  |  |  |  |  |  |  |
| 12 months - 24 months | | 0.48 | 0.28 | 0.087 |  | 0.49 | 0.29 | 0.087 |  | 0.48 | 0.27 | 0.078 |
| 12 months - school age | | 0.25 | 0.23 | 0.266 |  | 0.23 | 0.23 | 0.320 |  | 0.85 | 0.22 | **< .001** |
| 24 months - school age | | -0.23 | 0.25 | 0.351 |  | -0.26 | 0.25 | 0.296 |  | 0.37 | 0.24 | 0.126 |

*Note*. HL-ASD, high likelihood diagnosed with autism; HL-noASD, high-likelihood not diagnosed with autism; LL, low likelihood.

BRIEF GEC, Behavior Rating Inventory of Executive Function Global Executive Composite. Flanker, Flanker Inhibitory Control and Attention Test. DCCS, Dimensional Change Card Sort.

Figure S1. Longitudinal trajectories of executive functioning with Flanker as outcome adjusted for covariates


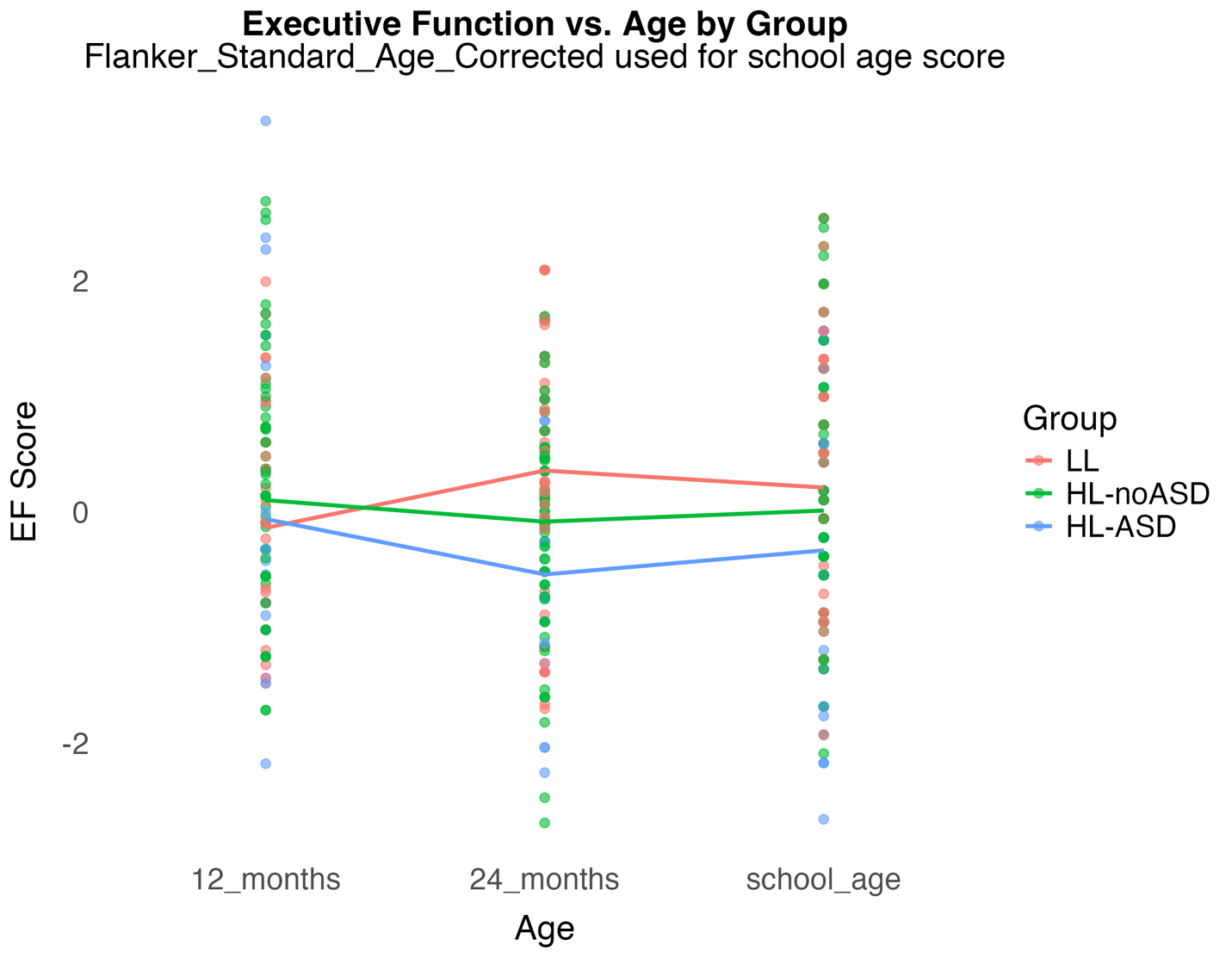


Figure S2. Longitudinal trajectories of executive functioning with DCCS as outcome adjusted for covariates


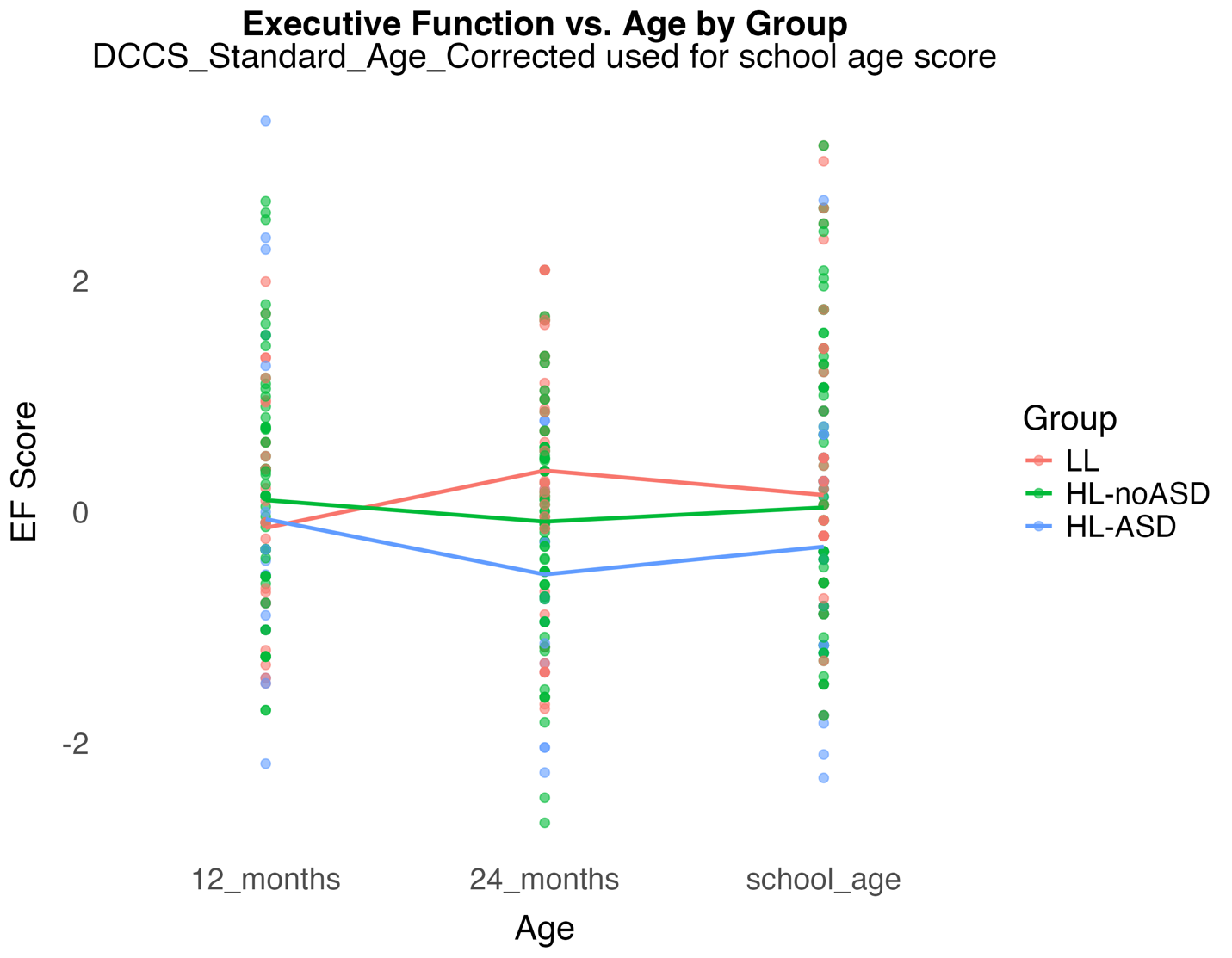


Figure S3. Longitudinal trajectories of executive functioning with BRIEF as outcome adjusted for covariates


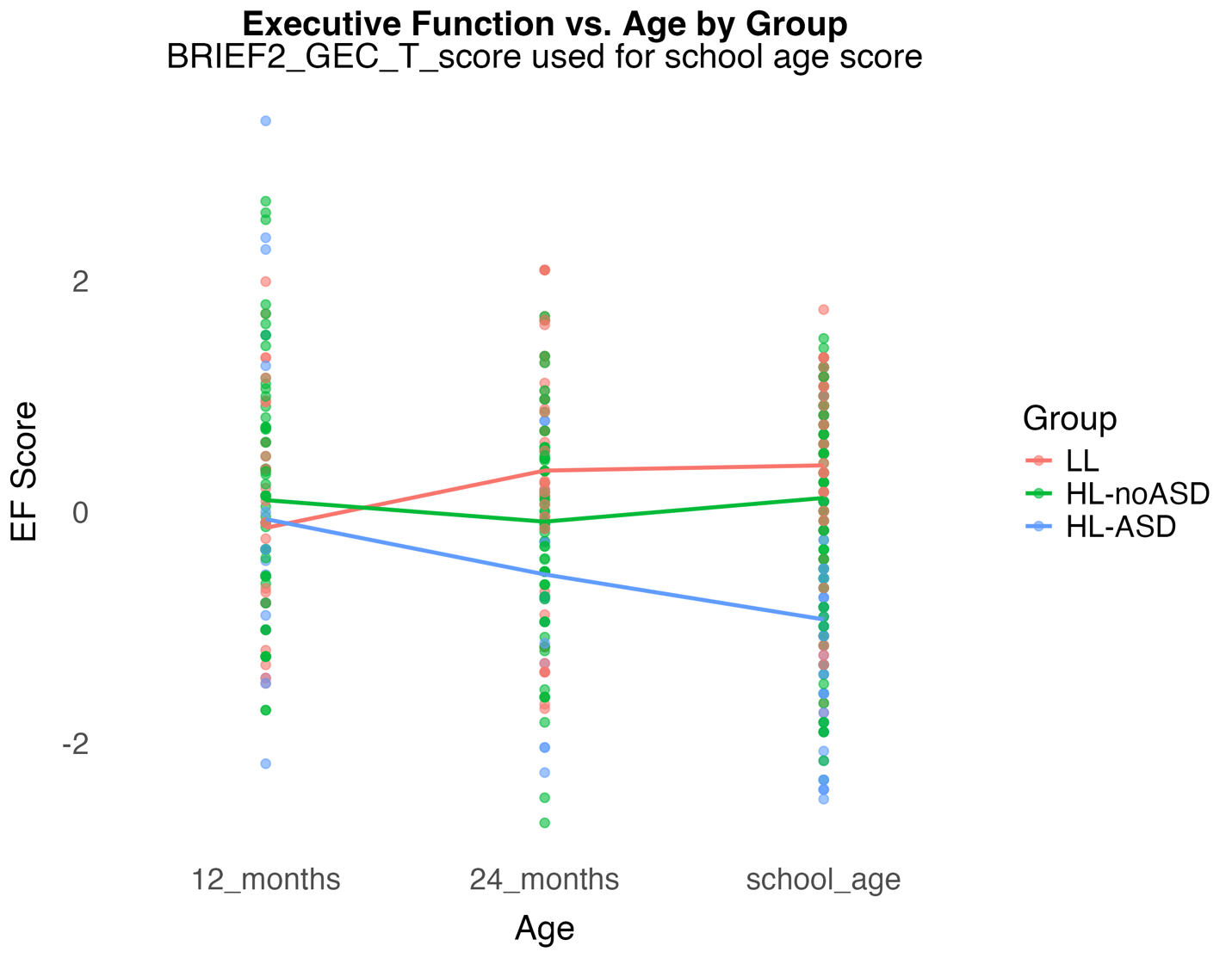


Figure S4. Longitudinal trajectories of executive functioning with BRIEF Working Memory as outcome


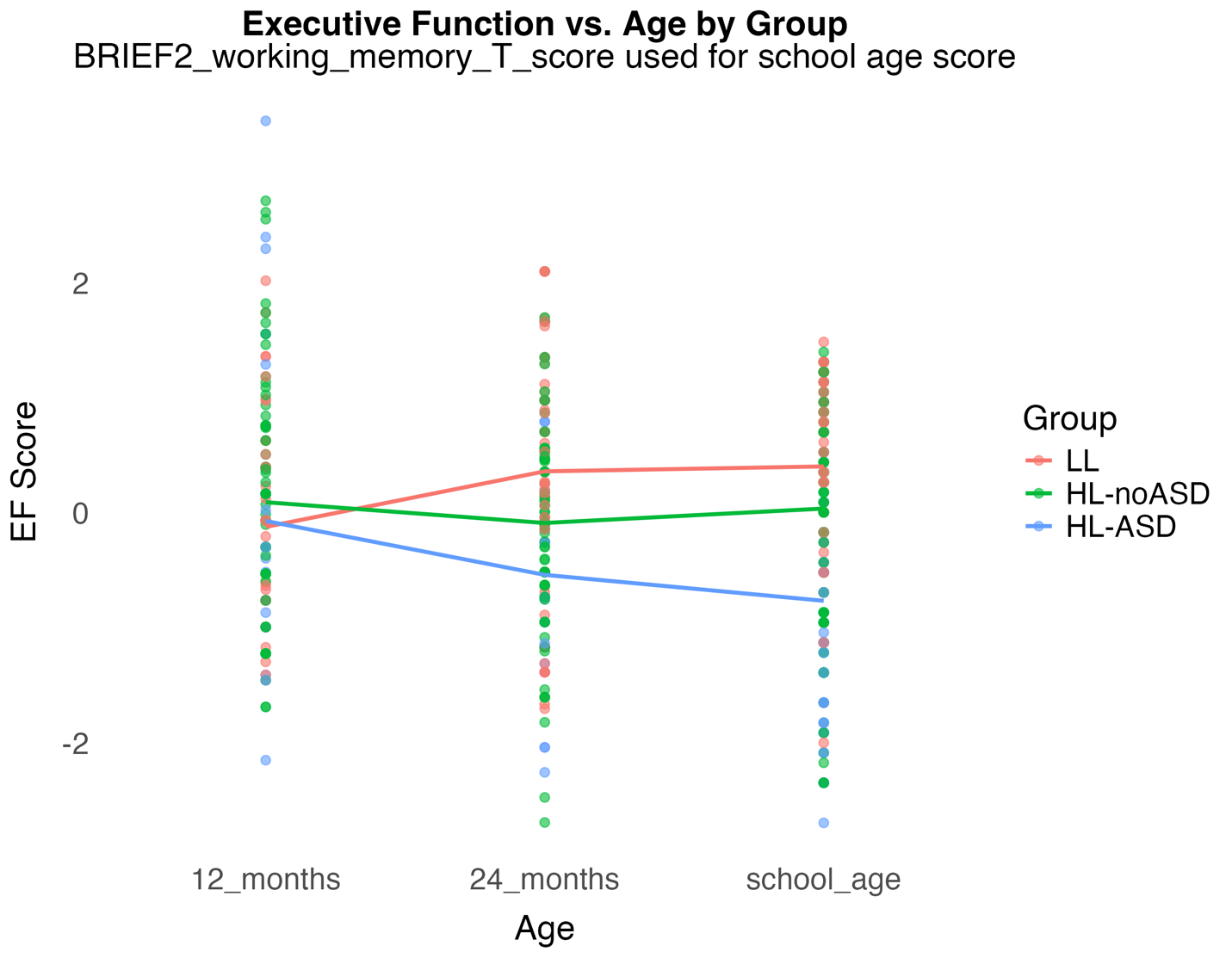


Figure S5. Longitudinal trajectories of executive functioning with BRIEF Inhibit as outcome


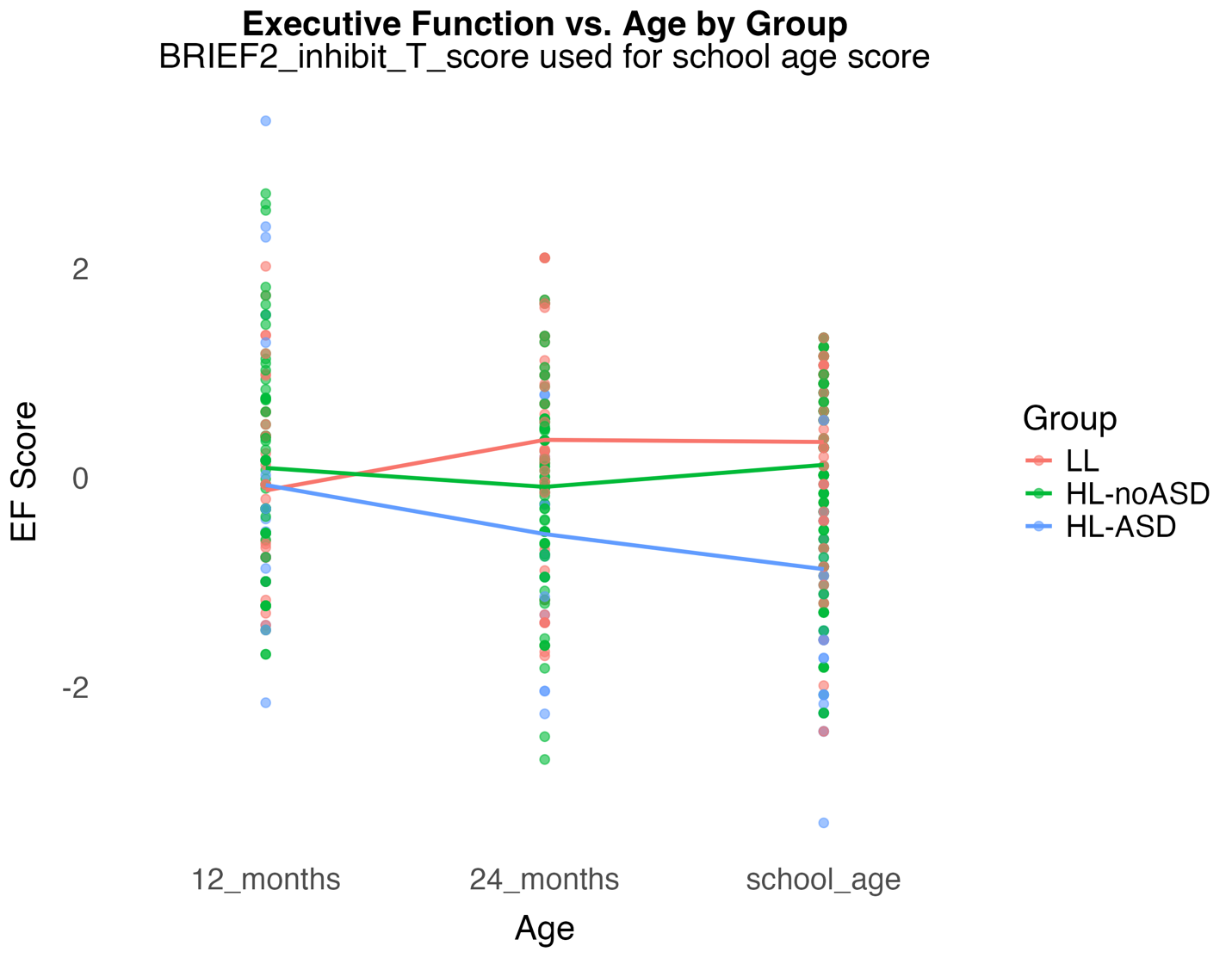


Figure S6. Longitudinal trajectories of executive functioning with BRIEF Shift as outcome


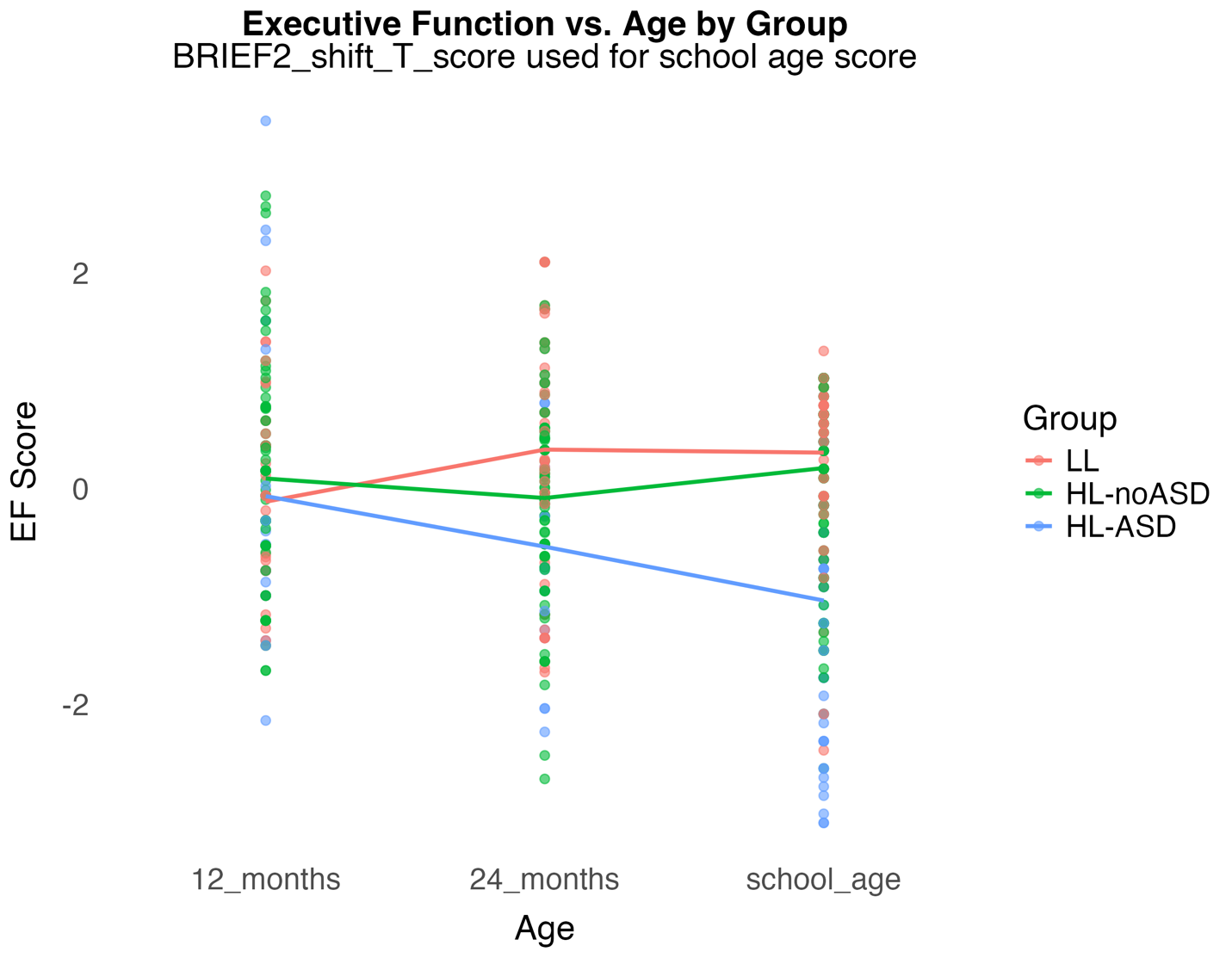

Supplement: Supplementary file 1 — Supplementary Material 1 [file 11689_2026_9682_MOESM1_ESM.docx]
